# An *in vitro* microbiological study comparing eight endotracheal tubes and their ability to prevent microaspiration

**DOI:** 10.1186/2197-425X-3-S1-A382

**Published:** 2015-10-01

**Authors:** M Mariyaselvam, L Marsh, M Wise, D Williams

**Affiliations:** University Hospital of Wales, Cardiff, United Kingdom

## Introduction

The major cause of ventilator-associated pneumonia (VAP) is the aspiration of bacteria-laden subglottic secretions past the cuff of the endotracheal tube (ETT) [[1]]. When the ETT cuff is inflated to the correct wall pressure, excess cuff material folds and causes involutions thereby forming channels, which allow leakage of subglottic secretions to the lungs [[2]]. Now, new ETT cuffs have been designed and subglottic ports added in order to prevent microaspiration. The PneuX tube has previously been shown to prevent leakage by eliminating cuff folds and other cuffs have shown variable improvements [[3]].

## Objectives

This study aimed to compare the properties of the new design ETTs against the PneuX and their ability to prevent leakage of a microbial contaminated solution.

## Methods

Seven endotracheal tubes were compared with the PneuX tube in *in vitro* studies. Using a sterile technique, the distal ETT was placed inside a sterile 2 cm 'static model trachea' and the cuff inflated according to manufacturers' instructions. A continuous cuff pressure monitor was used if indicated by the manufacturer. Four ml of bacterial suspension comprising of *Pseudomonas aeruginosa, Staphylococcus aureus* and *Candida albicans* was added above the ETT cuff. The time taken and the volume of the bacterial fluid to leak past the cuff was measured. This volume was collected, serially diluted, plated on to agar media and incubated aerobically 37°C for 24 h. The number of colony forming units (cfu/ml) were then determined. If an ETT did not leak, the experiment was terminated at 1 h and a phosphate buffer solution was injected into the space below the cuff and processed as above to determine microleaks. The experiment was repeated on 10 separate occasions for each type of ETT, with a new sterile ETT used each time.

## Results

**Figure 1:**
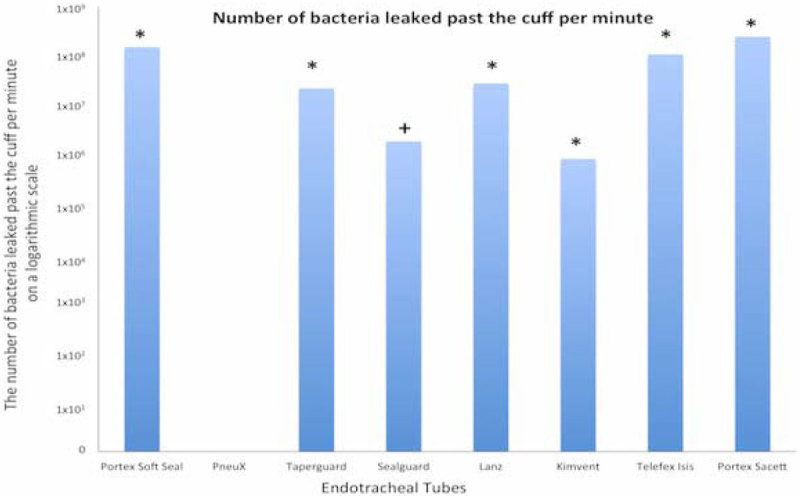
Showing mean number of bacteria leaked past the cuff per minute. Graph shows zero leakage of bacteria past the PneuX tube compared with all the other tubes (P < 0.05,*), except the Sealguard (P = 0.09, +).

Results were analyzed with a Fisher's exact test comparing the PneuX tube to the other ETT with multiple analyses and a Bonferroni correction. Figure 1 summarizes the results, which revealed a statistically significant elimination of bacterial leakage with the PneuX ETT compared to all other ETT (P < 0.05) with exception of the Sealguard (P = 0.09).

## Conclusions

To reduce bacterial contamination of the lungs and prevent VAP, it is necessary for the ETT to prevent aspiration past the cuff. The PneuX was the only ETT that consistently and completly achieved this goal in this *in vitro* study.

## Grant Acknowledgment

Funded by the Eastern Academic Health Science Network
